# Effect of the nanofilm-coated zirconia ceramic on resin cement bond strength

**DOI:** 10.34172/joddd.2022.029

**Published:** 2022-11-15

**Authors:** Viviane Maria Gonçalves de Figueiredo, Alecsandro de Moura Silva, Marcos Massi, Argemiro Soares da Silva Sobrinho, José Renato Cavalcanti de Queiroz, João Paulo Barros Machado, Renata Falchete do Prado, Lafayette Nogueira Junior

**Affiliations:** ^1^Department of Dental Materials and Prosthodontics, Institute of Science and Technology of São José dos Campos, São Paulo State University (UNESP), Sao Jose dos Campos, Brazil; ^2^Department of Physics, Technical Institute Aerospace (ITA), Sao Jose dos Campos, Brazil; ^3^Department of Biotechnology, UnP – Laureate University, Natal, Brazil; ^4^Laboratory of Sensors and Materials (LAS), National Institute of Space Research (INPE), São José dos Campos, Brazil

**Keywords:** Bonding, Fluorine, Nanofilm, Shear Test, Silicon, Zirconia

## Abstract

**Background.** New surface treatments have been proposed to expand the clinical indications of zirconia prostheses. This study aimed to evaluate the effect of silica and fluorine nanofilms on zirconia ceramic on the resin cement bond strength.

**Methods.** Zirconia blocks and discs underwent different surface treatments: untreated zirconia (CON), sandblasted, silica-coated alumina particles (30 µm) (SC), silica nanofilm (SN), and fluorine nanofilm (FN). Nanofilm deposition was performed through plasma enhanced chemical vapor deposition (PECVD). Zirconia surfaces were characterized on disks by morphology (atomic force microscopy, AFM), chemical analysis (x-ray photoelectron spectroscopy, XPS), and contact angle analysis. A silane coupling agent was applied on each treated surface, and a cylinder of resin cement was built up. Half of the specimens in each group were submitted to 6000 thermal cycles (TC). Bond strength was analyzed using the shear test, and the fractographic analysis was performed with stereomicroscopy and SEM/EDS. Statistical analysis was performed through one-way ANOVA and Tukey test in the non-aged and aged specimens.

**Results.** Nanofilms modified the zirconia surface, which became more hydrophilic and chemically reactive. Chemical bonding between Si-O was found in SN, and FN promoted a fluorination process on the ceramic surface, converting zirconia into zirconium oxyfluoride. Specimens of the SN (TC) group failed on pre-testing. FN (TC) bond strength (3.8 MPa) was lower than SC (TC) and CON (TC) after shearing. Adhesive failure predominated in the experimental groups. Silica nanofilm failure occurred after aging.

**Conclusion.** Silica and fluorine nanofilms deposited by PECVD did not promote effective bonding between zirconia and resin cement.

## Introduction

 The yttrium-stabilized tetragonal zirconia polycrystal (Y-TZP) has been used in the infrastructure of fixed prostheses to replace missing teeth due to improved mechanical, biological, and esthetic properties.^1^ However, the use of zirconia as infrastructure has been limited where bonding is required due to low bond strength to resin cements.^2^

 The tribochemical method (mechanical bonding) is the most suitable technique for the surface treatment of Y-TZP. Sandblasting with silica-coated alumina particles is easily performed in clinical practice, increasing the bond strength to a resin cement surface.^3^ However, the bonding stability with resin cements using a bonding agent on the silicatized zirconia is lower than silane on glass ceramics.^4^ Several factors may influence this result, such as the irregular silica deposition on the substrate, which limits the area available for chemical bonding of the silane agent and the development of cracks that may interfere with the mechanical properties, causing premature restoration failure.^5^

 New surface treatments have been proposed to expand the clinical indications for zirconia, including methods of Y-TZP surface modifications through nanofilms or plasma deposition.^6-8^ Nanofilms improve hydrophilic ceramic surfaces with increased surface energyand bond strength.^9^ The most commonly used nanofilm deposition methods are physical (PVD – physical vapor deposition) or chemical (PECVD – plasma enhanced chemical vapor deposition). Studies with PECVD presented the deposition of hexadimethylsiloxane (C_6_H_18_OSi_2_)^10^ and chloro-silane (SiCl_4_) on zirconia^8^ and obtained increased bond strength values between zirconia and resin cement.

 Fluorination processes were used in a few studies^11-13^ as an alternative treatment for zirconia surface modification. The oxygen fluoride chemically modifies the zirconia surface, causing it to become more reactive and hydrophilic, with ~7.8° contact angle,^11^ increasing bond strength on resin cement.^14^ The exact mechanism by which the fluorination improves chemical bonding with the silane agent is still unknown, requiring new experiments addressing the chemical mechanism of bonding and its use as a surface treatment in the long term.^11^

 This study aimed to evaluate the effects of silica and fluorine nanofilms deposited by PECVD in the bond strength between zirconia and resin cement. The null hypothesis was: There is no significant difference in the bond strength tests between the silica-coated group and fluorine nanofilm group after adhesive interface aging.

## Methods

###  Y-TZP specimen preparation

 The sample size calculation was performed via statistics software (Minitab version 16.1 for Windows, Pennsylvania, USA), based on the standard deviation of the study of de Queiroz et al.^15^ A significance level of 5%, a beta power of 80% using the bond strength the standard deviation of the outcome,^15^ a total of 8 samples were determined per group.

 Zirconia blocks for CAD-CAM (Y-TZP cubes, Vita Zahnfabrik, Germany) were prepared using a proper cutting device (ISOMET 1000, Buehler Ltd., IL, USA), generating 88 specimens measuring 15.2×12.5×1.7 mm for bond strength testing and zirconia surface characterization. Zirconia discs measuring 11.0 mm in diameter and 1.4 mm in height were prepared for contact angle analysis (n = 12).

 The specimens were sintered in a Zyrcomat furnace (Vita Zahnfabrik, Germany) according to the manufacturer’s instructions. Next, surface standardization was performed with 400-, 600-, 800-, and 1.200-grit wet silicon carbide paper (3M, Sumaré, Brazil) under constant water irrigation (AutoMet 250, Buehler Ltd., IL, USA). The specimens were then ultrasound-cleaned (4820 Digital Ultrasonic Clear, Blazer, NY) using 10% residue-removing isopropyl alcohol (Sigma-Aldrich, Darmstadt, Germany) for 4 minutes. Finally, the specimens were left to dry at room temperature, and surface treatment was performed.

 The specimens were divided into four groups (n = 20) according to the surface treatment: CON group: Y-TZP ceramic was not surface-treated; SC group: Sandblasting with silica-coated alumina particles (30 µm); SN group: Silica nanofilm; FN group: Fluorine nanofilm.

 In the SC group, the zirconia surface was sandblasted for 10 seconds at 2.8 bar, with silica-coated alumina particles (30 µm) (Rocatec^TM^ Soft, 3M ESPE AG, Seefeld, Germany) using a proper etching device (ERC MicroEtcher, Danville, San Ramon, USA) coupled to a metallic tip at 45°.^16^ The distance between the specimens’ surface and the metallic tip was standardized at 10 mm.

 In the SN group, nanofilm was obtained through the PECVD method, using hexadimethylsiloxane (C_6_H_18_OSi_2_) (Sigma Chemistry-Aldrich, Munich, Germany) as an initiator gas and a plasma reactor (Plasma and Process Laboratory from Technical Institute Aerospace - ITA, São Jose dos Campos, Brazil). The PECVD process was dry (0% moisture) as it was carried out in a vacuum chamber using ultra-high-purity gases (99.999%), and the temperature remained<100^o^C to avoid damage to the fluorine-based interface. Initially, pre-treatment with argon plasma (Ar) was performed on the specimens with a power of 250 W, 42.4 mTorr, a 10.3 sccm flow, and Vbias of -879 volts for 10 minutes. After concluding this step, the vacuum system/reactor pressure was 21.5 mTorr. Then, for nanofilm stability, hexadimethylsiloxane, oxygen gas (O_2_) (25 sccm), and argon gas (10.3 sccm) were deposited. The system pressure was 78.4 mTorr, with a power of 250 W and Vbias of -868 volts for 10 min. After 10 minutes, the reactor was opened to establish atmospheric pressure. The silica nanofilm thickness was 233.34 nm.

 In the FN group, the sulfur hexafluoride (SF_6_) (White Martins Gases Industrial SA, Rio de Janeiro, Brazil) was the initiator for the fluorine nanofilm growth using the same PECVD described above. The argon plasma was used as pre-treatment with the same parameters. The pressure in the vacuum system/reactor after argon plasma was 22.3 mTorr. The argon gas (10.3 sccm flow), sulfur hexafluoride (10 sccm flow), and hydrogen gas (25 sccm) were released in the reactor; the system pressure was 79.0 mTorr, with 250 W and a Vbias of -843 volts for 10 min. Oxygen (25 sccm flow) was released into the reactor during the last 2 minutes. After 10 minutes, the reactor was opened to establish atmospheric pressure. The silica nanofilm presented a thickness of 113.44 nm.

###  Surface characterization

 Specimens of each surface treatment (n = 1) were analyzed using atomic force microscopy (AFM) (Model Multimode, Veeco, New York, USA) to observe surface morphology and roughness. Surface scanning was conducted in intermittent contact mode, using an antimony-doped silicon tip, which was moved over the surface of the study in the X, Y, and Z axes, covering 2×2-, 3×3-, and 10×10-μm areas, allowing to acquire 2D and 3D images. Then surface roughness was evaluated using quadratic roughness average (Rq) and absolute roughness average (Ra) values.

 The chemical characterization of the specimens of each surface treatment (n = 1) was performed with x-ray photoelectron spectroscopy (XPS) (VSW HA 100 - VSW (L TOA Instrument Scientific, Manchester, England). The hv (incident photon) energy adopted for this study was 1486.6 eV. The most intense level of zirconium (Zr) Zr_3_d, carbon (C) C_1_s, oxygen (O) O_1_s, aluminum (Al) Al_2_p, silicon (Si) Si_2_p, and fluorine (F) F_1_s were analyzed. The spectra were analyzed using Origin Microcal Software (Northampton, USA).

 The contact angle was measured with a goniometer (Advanced Goniometer, Model 500, Ramé-Hart Instrument Co., New Jersey, USA) in disc specimens (n = 3). A single drop of distilled water was poured over each sample. The angle in the ceramic surface was immediately measured with specific software (DROPimage Advanced v. 2.4, Ramé-Hart Instrument Co., New Jersey, USA).

 The scratch test was carried out on specimens with nanofilm only (n = 2), using a tribometer with a Rockwell indenter (CERT UMT-2 Atibaia, Brazil). The test was conducted with a 10-N load and an increasing and initial speed of 0.2 N/s, covering 10 mm of the specimen in 100 seconds, determining the critical load for nanofilm rupture through acoustic emission.

###  Bonding test 

 Before cementation, the specimens of each experimental group were salinized. A drop of Clearfil porcelain bond activator (Kuraray Medical Inc., Okayama, Japan) was mixed with the primer of Clearfil SE Bond (Kuraray Medical Inc., Okayama, Japan) and applied to the ceramic surface with a microbrush (KG Sorensen, São Paulo, Brazil) (n = 10). Silanization of the silicate ceramics improves adhesion with the resin, but some authors disagree with its efficacy in oxide ceramics. Currently, silane may be applied to the oxide ceramics alone or used in combination with MDP.^17^ A short 60-second period was given for the reaction of this material with the Y-TZP surface, and the excess was removed with compressed air at 2.8 bar for 15 seconds, according to the manufacturer’s instructions.

 Then, PANAVIA F resin cement (Kuraray Medical Inc., Okayama, Japan) was manipulated and inserted into a cylindric mold (3 mm in height and 3.5 mm in diameter) with a Centrix (Nova DFL, Rio de Janeiro, Brazil). Polymerization occurred in 40 seconds (Radii-Cal, SDI Limited, Australia) under a light intensity of 1200 mW/cm^2^). After curing, Oxiguard II (Kuraray Medical Inc., Okayama, Japan) was applied for 3 minutes. Next, it was removed with water according to the manufacturer’s recommendation.

 The specimens were immersed in distilled water at 37°C for 24 hours.

 Then, half of the samples were submitted to the thermal cycle (aging experiment). The groups were named: CON (TC), SC (TC), SN (TC), and FN (TC). The final number of samples was n = 10 per group for the bond strength test.

 The aged groups were submitted to thermal cycling. The specimens were exposed to 6.000 thermal cycles, with baths of 5 ± 1°C and 55 ± 1°C (Nova Ética, São Paulo, Brazil) and next subjected to shear bond strength testing.

 The specimens were embedded in chemically activated acrylic resin (Jet Clássico, São Paulo, Brazil) in a plastic cylinder and positioned in the universal testing machine (EMIC, São José dos Pinhais, Brazil). A knife-like tool was positioned on the load cell (50 kgf) of the testing machine, and the load was applied to the ceramic/cement interface at a constant speed of 1 mm/min until fracture occurred. The bond strength was calculated as R = F/A, in which “R” is the bond strength (MPa), “F” is the force applied to break the specimen (N) and “A” is the interfacial area of the specimen, πr^2^ (mm^2^). The standardized bond area was 9.62 mm.^2^

 The fractured samples were analyzed using a stereomicroscope (Discovery V20, Zeiss, Gottingen, Germany) under ×20 magnification, and fractures were classifiedas follows^18^:

- *Adhesive along with the ceramic/cement interface:* the ceramic surface presented no resin cement in the adhesive area or<1/3 of resin cement in the adhesive area
- *Cohesive cement:* the resin cement was partially fractured, leaving>2/3 of cement in the adhesive area
- *Mixed:* failure occurred at the adhesive interface, but 1/3 to 2/3 of the resin cement remained in the adhesive area

###  Fracture surface analysis

 Representative images of the fracture were analyzed through a scanning electron microscope (SEM) (Inspect S50, FEI Company, Orlando, USA). Representative images of the bond area from nanofilm groups were selected for the chemical identification with electron dispersive spectroscopy (EDS). The Bruker 1.9 Espiret program has an EDS detector attached to the SEM. The mapping was performed on the adhesive area of the specimen for 5 minutes. The elemental concentration was determined after calculating the average percentages of chemical elements per weight.

###  Statistical analysis

 Data analysis was performed using Minitab 16.1 for Windows (Pennsylvania, USA). One-way ANOVA and multiple comparisons Tukey tests (post hoc tests) were used to compare the experimentally aged and non-aged groups. Student’s *t* test was performed to evaluate the aging effect within each surface treatment type. A significant level of 5% was adopted.

## Results

###  Surface characterization

**Figure 1:**
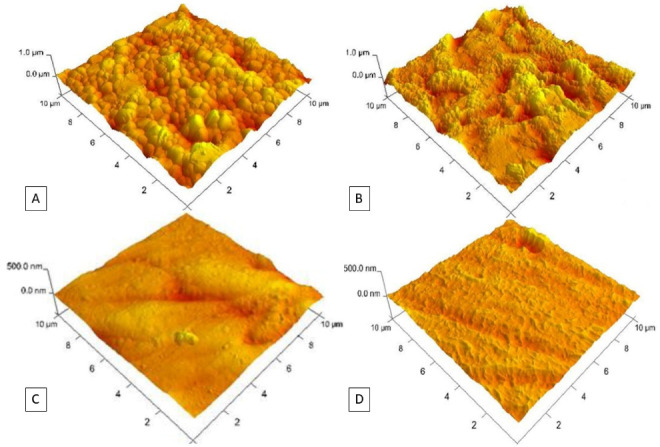
AFM photomicrographs of zirconia with different surface treatments: CON (A); SC (B); SN (C); FN (D).

Table 1 presents roughness, contact angle, and scratch test results. The AFM revealed the coating surface topography of the Y-TZP (Figure 1A), sandblasted (Figure 1B), the silica nanofilm (Figure 1C), and the fluorine nanofilm surfaces (Figure 1D). The presence of nanofilms changes the surface topography. The zirconia grains visible in the control group were not observed after nanofilm deposition (Rq 42.7). The topography of a sandblasted surface (Rq 106.0) with peaks and valleys showed a rougher surface compared to the other groups (Table 1).

**Table 1:** Surface treatment, roughness (nm) values, mean of contact angle (°), and standard deviation (SD) of scratch test (N) values

**Surface** **treatment**	**Roughness**		**Contatct angle**	**Scratch test**
	**Rq**	**Ra**		
CON	42.7	53.8	69.2 (8.8)	-
SC	106.0	82.6	17.2 (5.6)	-
SN	15.2	20.1	21.8 (1.0)	4.0
FN	34.2	25.0	8.0 (0.4)	15.0

 Silica nanofilm-treated zirconia demonstrated lower roughness values (Rq = 15.2) than the non-treated zirconia surface. The surface with fluorine nanofilm treatment had higher roughness (Rq = 34.2) than silica nanofilm but smoother than the untreated group (Table 1).

**Figure 2:**
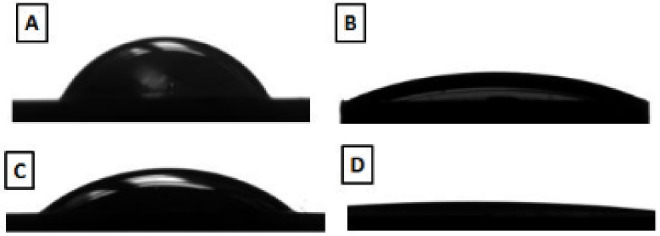
Drop on zirconia with different surface treatments: CON (A); SC (B); SN (C); FN (D).

 The critical load in silica nanofilm was lower than in the fluorine nanofilm group in the scratch test (Table 1). After nanofilm deposition, the Y-TZP surface became more hydrophilic. FN showed a lower contact angle than SC and SN (Table 1 and Figure 2).

**Figure 3:**
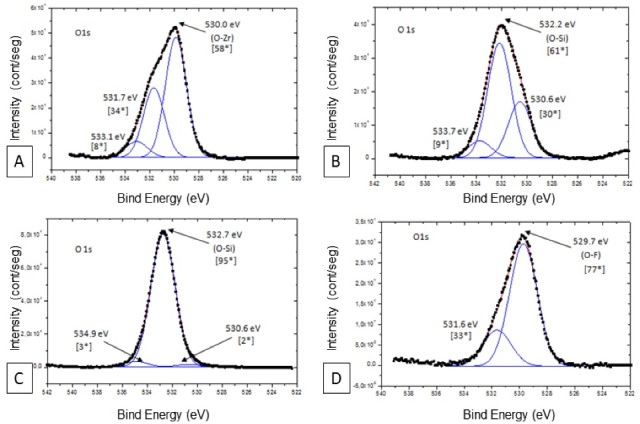
XPS ESCA chemical analysis. Oxygen spectrum (O_1_s) with different surface treatments: CON (A); SC (B); SN (C); FN (D).

Table 2 presents the main energy spectrum peaks obtained in XPS analysis. In group SN, the O_1_s spectrum had three peaks, but the energy peak of 532.7 eV demonstrated predominance. This peak corresponded to O-Si (SiO_2_) energy. This energy peak showed a chemical change in the ceramic surface; i.e., the half-height width (2.3) and relative intensity (95*) were higher than those found in sandblasted samples (61*). In the untreated zirconia, the O_1_s spectrum also had three peaks, but the energy peak of 530.0 eV demonstrated predominance. This peak corresponded to O-Zr energy (Figure 3). In the FN group, the predominance was demonstrated in the energy peak of 529.7 eV, with the energy of O-F (77*).

**Table 2:** Surface treatment and binding energy (eV)

**Surface** **treatment**	**Bind energy**					
	**O**_1_**s**	**C**_1_**s**	**Al**_2_**p**	**Si**_2_**p**	**F**_1_**s**	**Zr**_3_**d**
CON	530.0	284.6	-	-	-	182.0
SC	532.0	284.6	74.0	102.3	-	182.4
SN	532.7	284.6	-	103.4	-	-
FN	529.7	284.6	-	-	684.6	182.2

 All the groups demonstrated the main peak of C_1_s at 284.6 eV. The carbon relative intensity (88*) was higher in the SN treatment.

**Figure 4:**
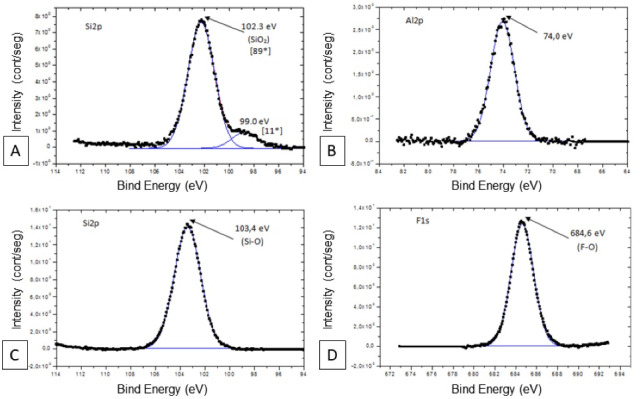
XPS ESCA chemical analysis. A) SC silicon spectrum (Si_2_p). B) SC aluminum spectrum (Al_2_p); C) SN Silicon spectrum (Si_2_p); D) FN fluorine spectrum (F_1_s).

 The silicon was found in the sandblasted group and silica-based nanofilm. In group SC, the Si_2_p spectrum had an energy peak of 102.3 eV and demonstrated predominance. This peak corresponded to SiO_2_ energy, with relative intensity (89*). In group SN, the Si_2_p spectrum presented the energy peak of 103.4 eV, corresponding to SiO_2_ energy (Figure 4A and 4C).

 Fluorine was observed in F_1_s spectra only in the FN group (Figure 4D). According to the spectrum analysis, there was evidence of oxygen fluoride (F_y_O_x_) formation on the ceramic surface in the FN group. The peak of F_1_s spectra was observed at 684.6 eV. Aluminum was demonstrated, in Al_2_p spectra, at a peak of 74.0 eV, only in the SC group (Figure 4B).

**Figure 5:**
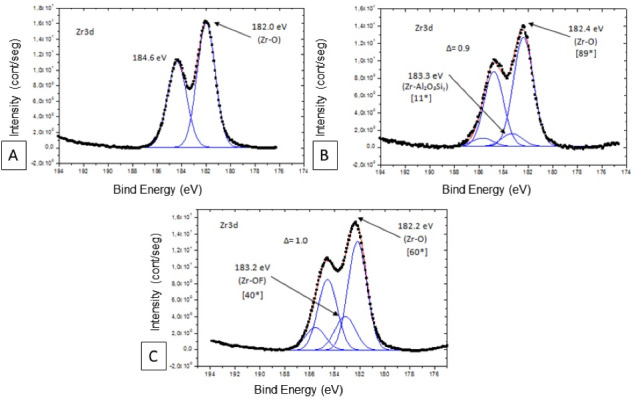
XPS ESCA chemical analysis. Zirconia spectrum (Zr_3_d) with different surface treatments: CON (A); SC (B); FN (C).

 Zr_3_d spectrum was not captured in the SN group. FN treatment changed the behavior of the zirconia spectrum, comparing the Zr_3_d spectrum of the untreated zirconia (Figure 5A), confirming the ceramic surface modification by plasma deposition with a more reactive structure. This surface modification after plasma fluorination was evidenced by the different spectrum presentations, potential energy (Δ = 1.0), and increased energy (182.2 eV - 183.2 eV) (Figure 5C), which were like the energy shown by sandblasted zirconia (Δ = 0.9, 182.4 eV - 183.3 eV) (Figure 5B). However, the relative intensity of the Zr-OF bond was not higher than the Zr-O bond shown in the Zr_3_d spectrum.

###  Bonding test 

 Adhesive-type failures were prevalent among all the experimental groups. The bond strength values decreased in aged groups compared to the non-aged groups, demonstrating significant differences, except in the SC group (Table 3).

**Table 3:** Comparison of the bond strength values of each surface treatment before and after aging and *P* values

**Compared groups**	***P***** value**
CON vs. CON (TC)	0.016*
SC vs. SC (TC)	0.107
FN vs. FN (TC)	0.001*

*The groups were significantly different (Student’s *t *test comparison, α = 0.05) for bond strength data with and without aging.

 The SN (TC) group was lost during aging, as well as two specimens in the FN (TC) group (Table 4). The SC group demonstrated significantly higher values of bond strength when compared to the CON and FN groups. The CON and FN groups did not differ from each other. The same behavior of data was observed with and without the aging experiment (Table 4).

**Table 4:** Experimental groups, aging, number and percentage of failures during aging, number and percentage of adhesive, cohesive and mixed failures, mean (MPa) and standard deviation (SD) of adhesive failures, and *P* values for comparison

**Groups**	**Aging**	**Failures during aging**	** Failure types **			**Bond strength**	***P***** value**
			**Adhesive**	**Mixed**	**Cohesive**		
CON	No	-	8 (80.0%)	2 (20.0%)	0 (0.0%)	9.2^B^(2.3)	0.001*
SC	No	-	10 (100.0%)	0 (0.0%)	0 (0.0%)	19.0^A^(3.3)	
SN	No	-	10 (100.0%)	0 (0.0%)	0 (0.0%)	3.6^C^(1.0)	
FN	No	-	10 (100.0%)	0 (0.0%)	0 (0.0%)	11.1^B^(3.6)	
CON (TC)	Yes	0 (0.0%)	9 (90.0%)	1 (10.0%)	0 (0.0%)	6.3^a^(1.7)	0.001**
SC (TC)	Yes	0 (0.0%)	8 (80.0%)	0 (0.0%)	2 (20.0%)	15.4^b^(4.9)	
SN(TC)	Yes	10 (100.0%)	_	_	_	_	
FN (TC)	Yes	2 (20.0%)	8 (100.0%)	0 (0.0%)	0 (0.0%)	3.8^a^(0.7)	

* The groups were significantly different (one-way ANOVA comparison, α = 0.05) for bond strength data, non-aged. ** The groups were significantly different (one-way ANOVA comparison, α = 0.05) for bond strength data, aged. ^A^Groups with the same letter presented no significant differences (Tukey test; α = 0.05) for bond strength data, non-aged. ^a^Groups with the same letter presented no significant differences (Tukey test; α = 0.05) for bond strength data, aged.

###  Fracture surface analysis

 The fractographic analysis identified the fracture type and confirmed the chemical elements present in the adhesive area through EDS analysis.

**Figure 6:**
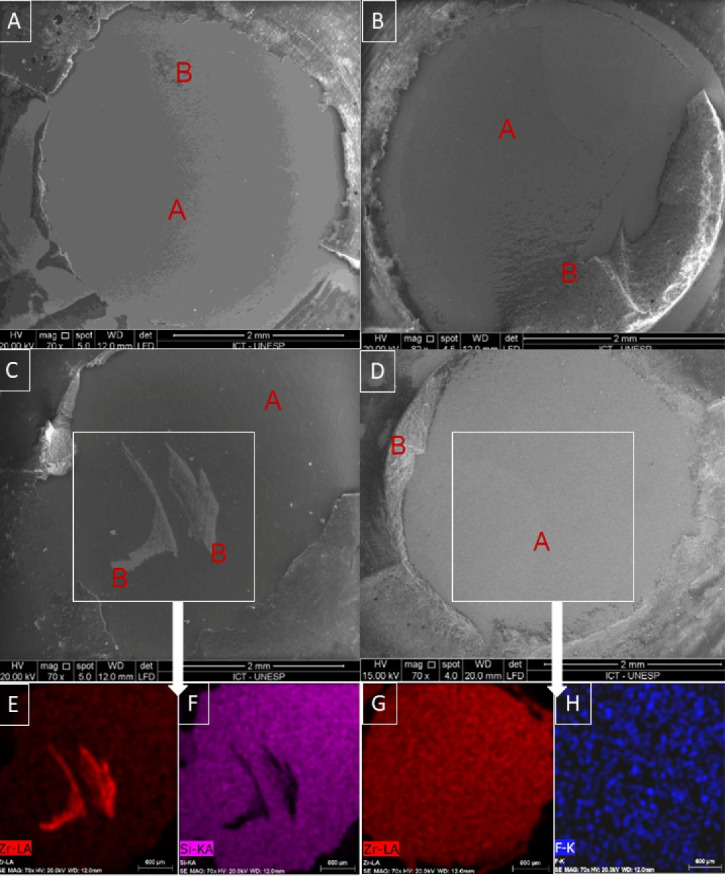
SEM and EDS photomicrographs of bonding area. (A) The CON (TC) group; point A demonstrates zirconia, and point B illustrates the residue of resin cement. (B) The SC (TC) group; point A demonstrates zirconia and aluminum, and point B illustrates the residue of resin cement. (C) The SN group; adhesive failure; point A represents silicon, and point B shows zirconia. (D) The FN group; point A demonstrates zirconia and fluorine, and point B illustrates the residue of resin cement. (E) and (F) Mapping the adhesive area of an SN group sample shows that silicon is present in the entire adhesive area (in purple), and zirconia is present only in points B, in which the detachment of the film occurred (in red). (G) and (H) Mapping the adhesive area of an FN group sample; it is possible to observe that zirconia is distributed in the adhesive area (in red), and fluorine is dispersed (in blue).

 The CON and CON (TC) groups exhibited predominantly adhesive failures (Figure 6A). The CS group demonstrated a prevalence of adhesive-type failures and, after thermocycling, exhibited 20% of cohesive failures (Figure 6B).

 The SN group only exhibited adhesive-type failures and detachment points of the silica nanofilm. Mapping the adhesive area by EDS enables the identification of the presence of zirconia and silicon (Figure 6C, E, and F).

 All the samples in the FN group (TC) exhibited adhesive failures. Mapping of the adhesive area by EDS demonstrated the dispersion of zirconia and fluorine throughout the adhesive area (Figure 6D, G, and H).

## Discussion

 This study simulated the adhesive performance between treated surfaces on the Y-TZP ceramic and resin cement with MDP, reproducing a clinical scenario with a ceramic restoration cemented with resin cement. Therefore, it is important to extend the results to clinical practice and dentists’ knowledge. The field of research for developing a reliable protocol for optimum zirconia bonding is still open.^19^ Herein, we aimed to explore the PECVD technique to obtain different nanofilms and test its potential to enhance the bond strength between Y-TZP and PANAVIA F resin cement. We successfully deposited silicon-based and fluorine-based nanofilms on the Y-TZP surface, changing its topography, roughness, and hydrophilicity.

 Zirconia blocks sintered as recommended by the manufacturer without any surface treatment were used as the control group. Sandblasting using 30-µm silica-coated alumina particles has been shown to enhance the adhesion of resin cements to Y-TZP and augment the fatigue limit by 15–31%^20^; therefore, the sandblasted group (SC) was used as a gold standard.^19^

 Experimental nanofilm groups demonstrated enhanced wettability on the Y-TZP surface. Even though surface modification influences the contact angle, the hydrophilicity did not improve bond strength. The silicon-based nanofilm group exhibited the lowest bond strength value without aging, and all resinous cement blocks detached from Y-TZP samples in this group during the thermal cycling of the aging experiment, consistent with a previous study that also reported pre-test failures in zirconia coated with the silicon-based film.^6^

 Aging has negative effects on adhesion to zirconia.^19^ Thermocycling causes repeated thermal expansion and contraction of the materials, possibly causing fatigue at the interphase and reducing bond strength values. Combining the two mechanisms of bond weakening procedures (hydrolysis and fatigue) results in a higher decrease in bond strength.^21^ The detachment might be explained because the experimental nanofilm groups demonstrated decreased roughness. Increased surface roughness provides a more extensive area for adhesion.^19^

 To deeply explore the difference in chemical affinity of each nanofilm deposited over zirconia, we employed XPS in this study. In the SN group, the formation of silica was confirmed on the zirconia surface after plasma deposition through energy peaks, suggesting a chemical bond between silicon and oxygen.

 The fluorination with 10-methacryloyloxydecyl dihydrogen phosphate monomer (MDP) containing PANAVIA SA Plus resin cement increased the resin bond strength to zirconia.^22^ It seemed to be promising data; therefore, herein, we decided to deposit a fluorine-based nanofilm on the Y-TZP surface. Even though the FN treatment resulted in higher roughness values than the SN group, it had low bond values, being lower than the gold standard surface treatment with sandblasting with silicon-coated alumina. Accordingly, FN exhibited 20% of pre-test failures in the aging experiment compared to SC and CON groups, demonstrating no failures during aging.

 In the FN group, the nanofilm promoted a process of fluorination on the zirconia, converting the Y-TZP (ZrO_2_) to zirconium oxyfluoride (ZrO_x_F_y_) or zirconium fluoride (ZrF_y_), favoring surface reactivity for acid-base reactions.^23^ This justifies the zirconium spectrum chemical modification compared to untreated zirconia. The single high fluorine spectrum energy peak explains the intense chemical reactivity generated by this surface treatment.

 In XPS, the main oxygen spectrum energy peak was similar in the CON and FN groups. The bonding energy reduction in fluorine nanofilm in the oxygen spectrum is suggestive of the replacement of oxygen by fluorine to form zirconium oxyfluoride.^23^ Sulfur compounds, Zr(SO_4_)_x_, and Zr(SO_4_)_x_F_y_ are possible subproducts formed during the deposition process due to sulfur hexafluoride use (SF_6_). Attention must be paid to modifications in the underlying YSZ substrate after plasma fluorination, adversely affecting its outstanding mechanical and physical properties.^23^

 The lack of zirconium identification in the SN group can be explained by the fact that XPS analyzes the most superficial portion, and under these conditions, only the 233.34-nm thickness of nanofilm was accessed, which did not favor zirconium spectrum observation.

 The increased bond energy and relative intensity of oxygen occurred due to plasma reaction parameters during the deposition of silicon-based nanofilm. The high relative intensity of carbon in silica nanofilm was attributed to the hexadimethylsiloxane (C_6_H_18_OSi_2_), the initiator gas, which is carbon-composed. Thus, the plasma nanofilm growth does not deposit silica nanofilm alone, but a carbon-composed nanofilm is also deposited. The PECVD method includes complex chemical reactions, which involve subproduct formation and deposition of multiple components.^24^

 As reported in previous studies, the bond strength values obtained with nanofilm specimens were not higher than those obtained with silica-coating.^8,14,25^ These results may be explained by different deposition parameters, precursor gases for silica nanofilm, and subproduct formation.^23^ Bond strength to silica nanofilms was like those found in the study of Derand et al,^10^ who also used the hexadimethylsiloxane (C_6_H_18_OSi_2_) as an initiator gas in the process. Unfortunately, the bond strength of experimental nanofilms (FN and SN) was lower than expected. The thickness of nanofilms disfavored bonding to resin cement. The detachment after aging was also found by Queiroz et al,^7^ and it occurs probably due to nanofilm thickness limitations.

 We believe that water from the bath might contribute to the zirconia–cement bond degradation. Water penetration into the interface area might cause hydrolytic degradation of the resin cement matrix since all the aged groups exhibited lower bond strength values. No matter which factor (thermal or hydrolytic) is dominant, water undoubtedly plays a key role in weakening the bonding durability of zirconia.^26^

 The hypothesis presented in this study was not confirmed. We suggest further studies to test the effectiveness of plasma nanofilms on zirconia in enhancing resin cement bond strength, considering attempts to decrease nanofilm thickness and promote more efficient deposition processes with different gas initiators to obtain reduced formation of subproducts. Also, we believe that including groups without the silane/adhesive pre-treatment to improve chemical characterization with the silane/adhesive pre-treatment followed by solvent washing will help explain outcomes.

## Conclusion

 Silica and fluorine nanofilms deposited by PECVD did not promote bonding between zirconia and resin cement.

## Author Contributions

 VMGF, RFP, AMS, and JPBM contributed to conceptualization, data curation, data analysis, methodology, and validation. MM, AMS, JRCQ, and ASSS contributed to project administration, resources, and writing. All authors read and approved the final manuscript.

## Acknowledgments

 The authors thank Professor Richard Landers and the Department of Applied Physics, Institute of Physics, “Gleb Wataghin” (Unicamp), Campinas, Brazil that contributed to the characterization analysis (XPS).

## Funding

 FUNDUNESP Fundação para o Desenvolvimento da UNESP (“Júlio de Mesquita Filho” São Paulo State University Development Foundation) São Paulo, Brazil, 91431/13-DFP process.

## Ethics Approval

 Not applicable.

## Competing Interests

 The authors declare no conflict of interest.
